# Leveraging knowledge for explainable AI in personalized cancer treatment: challenges and future directions

**DOI:** 10.3389/fdgth.2025.1637195

**Published:** 2025-09-29

**Authors:** Emilia Daghir-Wojtkowiak, Javier Alfaro, Michele Mastromattei, Aleksander Palkowski, Mark Stares, Ana Roca-Umbert, Andraz Krajnc, Riccardo Leoni, Anne Boland, Aria Nourbaksh, Ashwin Kallor, Camille Ducki, Davide Venditti, Carla Montesano, Chiara Cipriani, Daniel Faria, Delphine Pflieger, Elisa Zago, Etienne Bardet, Filipa Serrano, Florian Jeanneret, Damien Alouges, Liangwei Yin, Elodine Coquelet, Apolline Bacquet, Francesco Bonchi, Francesco Maiorino, Francesco Torino, Georges Bedran, Jean-Alexandre Long, Laura Balbi, Laurent Guyon, Liana Bevilacqua, Manuel Fiorelli, Marie-Catherine Wagner, Mario Reyes, Mario Roselli, Marta Contreiras Silva, Michal Waleron, Nikolas Dovrolis, Odile Filhol-Cochet, In Hwa Um, Georg Wolflein, Patrícia Eugénio, Pauline Bazelle, Pavlos Golnas, Peter Thorpe, Pierluigi Bove, Piyush Borole, Roberta Bernardini, Rohit Kumar, Rosella Cicconi, Saskia Kaltenbrunner, Saverio Gravina, Simona Brezar, Stefan Symeonides, Steven McGinn, Susana Nunes, Ted Hupp, Yuri Gordienko, Dimitrios Varvaras, Sergii Stirenko, Luciano Xumerle, Stefania Mariani, Assilah Bouzit, Stéphane Gazut, Heiko Poth, Kyriakos Souliotis, Hector Katifelis, Elena Verzoni, Giuseppe Procopio, Sarah Schoch, Francisco Lupiáñez-Villanueva, Sandra Türk, Katarzyna Barud, Dimitri Koroliouk, Juan Caubet, Yamir Moreno, Jean-Luc Descotes, Christina Golna, Valentina Guadalupi, Paolo Garagnani, Maria Gazouli, Jean-François Deleuze, Frans Folkvord, Nikolaus Forgó, David J. Harrison, Håkan Axelson, Armando Stellato, Maurizio Mattei, Ajitha Rajan, Alexander Laird, Christophe Battail, Catia Pesquita, Fabio Massimo Zanzotto

**Affiliations:** ^1^ International Centre for Cancer Vaccine Science, University of Gdansk, Gdansk, Poland; ^2^ University of Rome “Tor Vergata”, Rome, Italy; ^3^ Urology Department, Western General Hospital, NHS Lothian, Edinburgh, United Kingdom; ^4^ PredictBy Research and Consulting SLU, Barcelona, Spain; ^5^ Caretronic d.o.o. Kranj, Slovenia; ^6^ DSTECH S.r.l., Rome, Italy; ^7^ CEA, Centre National de Recherche en Génomique Humaine, Université Paris-Saclay, Evry, France; ^8^ Centre Hospitalier Universitaire de Grenoble (CHU), Grenoble, France; ^9^ LASIGE, Faculdade de Ciências, Universidade de Lisboa, Lisboa, Portugal; ^10^ IRIG, Laboratoire Biosciences et Bioingénierie Pour la Santé, UA2 INSERM-CEA-UGA, Université Grenoble Alpes, Grenoble, France; ^11^ Personal Genomics Srl, Verona, Italy; ^12^ Eurecat, Centre Tecnològic de Catalunya, Barcelona, Spain; ^13^ Fondazione IRCCS Istituto Nazionale dei Tumori (INT), Milan, Italy; ^14^ Department of Innovation and Digitalisation in Law, University of Vienna, Vienna, Austria; ^15^ Laboratory of Biology, Medical School, National and Kapodistrian University of Athens, Athens, Greece; ^16^ School of Medicine, University of St Andrews, St Andrews, United Kingdom; ^17^ Health Policy Institute (HPI), Maroussi, Greece; ^18^ School of Informatics, University of Edinburgh, Edinburgh, United Kingdom; ^19^ Institute of Genetics and Cancer, University of Edinburgh, Edinburgh, United Kingdom; ^20^ National Technical University of Ukraine Igor Sikorsky Kyiv Polytechnic Institute, Kyiv, Ukraine; ^21^ EURICE, Heinrich-Hertz-Allee 1, St. Ingbert, Germany; ^22^ Division of Translational Cancer Research, Department of Laboratory Medicine, Lund University, Lund, Sweden; ^23^ Open University of Catalonia, Barcelona, Spain; ^24^ Institute for Biocomputation and Physics of Complex Systems and Department of Theoretical Physics, University of Zaragoza, Zaragoza, Spain; ^25^ Department of Communication and Cognition, Tilburg School of Humanities and Digital Sciences, Tilburg, Netherlands

**Keywords:** personalized cancer treatment, knowledge graphs, explainability, AI, foundation models, clinical decision-making

## Abstract

Integrating multi-modal patient data to support personalized medicine has gained a lot of interest across different health domains over the past decade. Addressing this challenge requires the development and implementation of an informed, evidence-based AI-driven decision-support system continuously maintained and updated to align with the latest clinical guidelines. A key challenge to ensure its real-life adoption lies in translating the outcomes of complex AI-driven data integration and modeling into a form easily understood by the clinical audience. To ensure explainability, knowledge graphs have emerged as data models integrating multi-omics data sources and representing them as interconnected networks. Knowledge graphs offer a framework which AI models can progressively refine, highlighting the most influential features and relationships facilitating transparency of complex interactions and interdependencies. In this perspective we present major components and challenges upon developing a knowledge-based explainable AI system. Additionally, we showcase a current effort undertaken by the Knowledge at the Tips of your Fingers (KATY) consortium to develop the infrastructure for an explainable system supporting best treatment decision for a renal cancer patient.

## Introduction

Integrating multi-modal patient data—such as genetic information, expression profiles, imagining and molecular data—into a unified framework has gained a lot of interest in different health domains over the past decade (1, 2). The recent increase in computational power and algorithm performance boost, have made it possible to feed AI models with extensive patient-specific data, paving the way for the development of software solutions that enable more personalized diagnoses and treatments (3, 4). Crucial to achieving this vision is not only the development and implementation of disease-focused, knowledge-based decision-support system but also its continuous maintenance and regular update to align with the latest clinical practice guidelines (5). Up to now, several tools supporting the vision of precision medicine have emerged.

IBM Watson for Oncology (WFO) was the first knowledge-based system leveraging natural language processing (NLP) supporting evidence-based treatment decisions categorized as *recommended*, *for consideration*, or *not recommended* across seven types of cancer. Alongside the NLP-driven WFO, OncoDoc2 emerged as a decision-tree-based system designed to integrate clinical data from electronic health records (EHR), to suggest optimal treatments for non-metastatic breast cancer (6) Similarly, a guideline-based decision support system developed as part of the DESIREE European project aimed at creating web-based services for managing primary breast cancer (7, 8).

In the field of medical image analysis including x-rays, CT images, and MRI scans, deep learning techniques have significantly enhanced tumor detection, localization, assessment of muscle invasiveness and tumor grading. These advancements facilitated the distinction between disease subtypes enabling more effective treatment planning and better patient stratification (9–16). As an example, a 3D deep radiomics pipeline has been successfully used for analyzing CT scans of metastatic urothelial cancer patients, demonstrating capability of differentiating between disease, control and progression in response to immunotherapy (17).

While creating a straightforward decision support system is technically feasible, a key challenge lies in translating the outcomes of complex AI-driven big data integration and modeling into a form easily understood by the clinical audience and in parallel compliant with binding legal provisions. To address this challenge, efforts are focused on integrating medical knowledge, biological pathways, and clinical guidelines into knowledge-based models. Developing a knowledge graph (KG) and incorporating it into AI model architecture, may provide explainable visualizations of the AI model's complex reasoning enhancing AI-supported, knowledge-based diagnosis and treatment recommendations within a specific biomedical domain. In this perspective we present challenges upon developing a knowledge-based explainable AI system and describe a current effort undertaken by the KATY consortium to develop the infrastructure for ready-to-use explainable decision-support system that can be deployed in clinical reality.

## How knowledge-based models interplay with AI model

Knowledge-based models integrate various multi-modal biomedical data sources by developing ontologies, which are structured frameworks for organizing information that define the relationships between key concepts and categories within a specific domain. These ontologies establish a formalized vocabulary, hierarchy of terms, relationships and rules to describe large volumes of heterogeneous biomedical data. Examples include HPO (Human Phenotype Ontology) (18), UMLS (Unified Medical Language System) (19), NCIT (National Cancer Institute Thesaurus) (20) and OMIM (Online Mendelian Inheritance in Men) (21) which serve as semantic “building blocks” for biomedical knowledge models. These structered, hierarchical ontologies and knowledge bases enable the development and enrichment of the knowledge models by defining standardizing terms, concepts, and relationships. They support transparent mapping between patient data, phenotypes, genotypes, and clinical concepts, facilitating tracability and interpretability.

Building on this foundation, knowledge graphs can represent diverse data sources as networks of interconnected entities (nodes) and relationships (edges) capable of capturing dynamic, real-world interactions and associations (22, 23). When tailored to a specific biomedical domain, KGs enhance data accessibility, interoperability and integration, facilitating more efficient data analysis and further interpretation.

A key challenge in building KGs is the scattered and inconsistent nature of information across disease-specific domains. In the cancer field, despite significant efforts made by consortia to develop standardized datasets, information from various organizational levels of datasets is dispersed across studies. Unstandardized data repositories, evolving ontologies, and the need to continuously adapt to ever-changing clinical guidelines (24) adds huge complexity to harmonization and integration of diverse data sources.

Building cohesive and functional KGs for cancer research faces several challenges related to: (i) Scalability - developing KGs requires manual labor and expensive expert input, making it difficult to scale; (ii) Variation in Cancer Representation Across Biomedical Data Repositories - cancer descriptions in medical repositories often do not follow standardized naming conventions or alignment with clinical guidelines, complicating large-scale data harmonization which hinders its usability and interoperability; and (iii) Ambiguity in Distinguishing Cancer Types - symptoms, causes, and manifestations of various cancers often overlap, making precise categorization of subtypes challenging.

Despite those intricacies, we observe constant efforts in building KGs from biomedical literature and clinical records. The Human Diseases Network (HDN) and Human Symptoms-Disease Network (HSDN) (25) have demonstrated the utility of disease-centric KGs via exploring the intricate relationships between clinical symptoms and diseases through molecular interactions enabling exploration of disease taxonomy and pathogenesis. The Scalable Precision Medicine Open Knowledge Engine (SPOKE) (26) has linked various biomedical databases to integrate individual patient data providing more personalized and data-driven healthcare insights. More recently PrimeKG, has offered a comprehensive and multi-modal view of diseases incorporating disease-associated perturbations in the proteome, biological processes, molecular pathways as well as anatomical and phenotypic scales, environmental exposures, and a range of approved and experimental drugs along with their therapeutic actions (24). Unlike the broad disease- or drug-oriented KGs, the Genetic and Rare Diseases Information Center (GARD) (27) focuses exclusively on rare diseases, aiming to advance understanding of unmet clinical needs and evidence-based research. In addition, over a thousand biomedical ontologies are emerging in BioPortal (28) - a comprehensive repository covering various cancer fields such as breast, thyroid, and prostate cancer. Despite their availability, leveraging these ontologies effectively remains challenging. Many were developed with a specific perspective and purpose in mind tailored to specific research goals or projects, which may not align with other use cases or compatibility with new applications.

In addition to existing disease-focused ontologies, there is a growing interest in building stage- and grade-specific ontologies aimed at deeper conceptualizations of a specific disease, however this concept has not been explored much in biomedical domains. In addition, transferring the knowledge embedded in KGs for the identification and characterization of rare disease is also of interest as nowadays, research in the rare diseases field is oriented towards the collection and analysis of omics data in case-study scenarios. Adoption of the KG could interconnect between several studies, synthesizing state-of-the-art knowledge and providing greater explainability of rare diseases not only to clinicians and patients but also generally to the field.

Leveraging knowledge graphs can streamline the learning process of AI models enhancing their explainability and transparency. Consequently, this interplay between both components significantly boosts interpretability (29) as KGs offers a framework that AI models can progressively refine. Through iterative learning, neural network architectures which naturally form graph-like structures (as entities encoded within these networks are interrelated), refine ontologies and highlight the most influential features and relationships. In consequence, the refined KG incorporates new information while eliminating inconsistencies, redundancies and duplicates. This refinement process facilitates improved detection of patterns, correlations and identification of broader categories or clusters within the data that have not been immediately apparent.

The interplay between the KG and the AI model would definitely support more robust AI-driven insights and decision-making, and is especially important for developing explainable recommendation systems which should ideally be responsive and adaptive to real-time changes.

## Enhancing explainability and interpretability of AI models

When dealing with unstructured multi-modal data, black-box models with more free parameters are preferred for capturing complex relationships in labeling, clustering, and pattern recognition tasks. While these models enhance the overall understanding of a system and its outputs, they do not inherently provide explanations that human experts can understand and comprehend (30, 31). Ensuring explainability and interpretability remains one of the biggest challenges in deploying AI-based models in real-world applications. Different models of explainability such as text-based, visual, and feature relevance explanations, are possible and should be adjusted to domain-specific research (30, 32, 33).

Recent work by Anthropic [https://transformer-circuits.pub/2024/scaling-monosemanticity/index.html] demonstrated that neuron-level interpretability in large language models is becoming achievable through advanced scaling techniques. Their approach aims to enhance model transparency by identifying monosemantic units—individual neurons that consistently represent specific, human-interpretable concepts—enabling a form of parameter-level understanding previously inaccessible in deep neural networks. While this aspect is significant for foundational AI safety and transparency, its direct applicability to clinical settings remains limited. In the context of medical AI, the primary focus should shift away from internal model mechanisms and instead emphasize whether the system demonstrates clinically reliable, safe, and interpretable behavior at the input–output level. Consequently, traditional validation frameworks such as sensitivity, specificity, and outcome-based performance metrics seem to align more closely with established clinical paradigms and are more trusted by healthcare professionals. Anthropic's findings suggest that medical AI systems should prioritize behavior-centric evaluations which is more practical and trusted forms of interpretability in healthcare applications.

To date, one study has utilized the knowledge graph fed with multi-source clinical data including basic clinical data, disease history, medical test results, and other Diabetic Macular Edema-related factors to predict this disease (34). In the field of precision radiotherapy, Niraula et al. developed a clinical decision-support system leveraging knowledge-based AI-assisted decision-making in response-adaptive radiotherapy (ARCliDS). The system was designed to adjust optimal daily dosage of radiation being the first web-based software with GUI dedicated to assist knowledge-based response-adaptive radiotherapy with multi-omics data improving the outcomes in dynamic treatment regime (35). Lately, the xDECIDE system has been developed and tested on various types of cancer as a tool to support clinical decision making effectively closing the learning loop through continuous, multi-source feedback integration. Central to this approach is the incorporation of expert-derived treatment recommendations from virtual tumor boards, which are used to iteratively train and refine the AI-based model (xCORE). In addition to expert input, the system incorporates real-world clinical data, such as treatment decisions made by patients and physicians, longitudinal clinical outcomes such as tumor response, adverse events, biomarker trajectories, and survival. The outcome data provide a foundation for retrospective performance evaluation and model recalibration. Furthermore, the xDECIDE platform leverages aggregated real-world evidence from the XCELSIOR registry (“Xperience Clinical Evidence Led System for Investigating Outcomes in Real-World Oncology”) to refine its knowledge base and decision-making heuristics. This closed-loop framework links what was recommended, what was implemented, and how patients responded to dynamically update treatment rationales and recommendations as new data is available. This idea of closing the learning loop not only has the potential of enhancing the relevance and accuracy of AI-driven guidance but also strengthens transparency, explainability, and clinical trust (36).

Despite these efforts, a systematic analysis of how explanations should be structured within disease-specific domains remains lacking. For AI-assisted diagnostic and treatment recommendation tools, explanations must be clear, comprehensive and conceptually coherent. Only then can these models provide transparency and interpretability essential for building trust within the medical community and establishing domain-expert interpretation and inference that align with current medical knowledge. Due to data sparsity, we need to leverage knowledge from publicly available omics data repositories and build knowledgeable databases within the context of specific diseases. Only by this we can advance knowledge-driven model development and promote explainable AI in healthcare (37–39).

To the best of our knowledge, there is no study which has utilized a knowledge graph-based explainable AI pipeline that integrates foundation models of omics data: genomics, transcriptomics, proteomics, to enhance diagnosis and/or treatment decision-making within the sparse data context in a specific biomedical domain. This effort has been undertaken by the *Knowledge at the Tips of your Fingers* (KATY) consortium funded in the H2020-EU.3.1. - SOCIETAL CHALLENGES - Health, demographic change and well-being programme under a call “*AI for Genomics and Personalized Medicine*” aims at implementing AI-assisted strategies for enhancing the contribution of -omics studies in personalized medicine.

## Effort of the KATY consortium towards developing explainable AI system

The KATY project develops a non-linear expert system capable of utilizing incomplete multi-modal omics information to predict the optimal treatment scheme in patients with metastatic clear cell renal cell carcinoma (ccRCC).

ccRCC accounts for 75% of total RCC incidence which increases worldwide and accounts for 2% of cancer diagnoses, affecting approximately 10/100,000 in the United States and Western Europe (40). Current treatment choice is largely driven by clinical trial inclusion criteria and guideline recommendations from the European Association of Urology (41), European Society of Medical Oncology (42) and American Society of Clinical Oncology (43) recommending first line treatment with combination immune checkpoint inhibitors (ICI) and tyrosine kinase inhibitors (TKI) therapy, or dual ICI for intermediate or poor risk disease. Despite these recommendations and the resultant improved survival benefit, still the response to treatment varies from individual to individual, complete response remains relatively rare, some patients develop significant adverse reactions to treatment and most patients eventually progress requiring a change in treatment (44).

Therefore, the greatest challenge is not only to gather more molecular or clinical information, but rather to better use and process the information that has already been gathered and is publicly available through the incorporation of pre-existing or newly developed foundation models. Despite a progress in AI-assisted noninvasive characterization of kidney tumors using CT imaging, better characterization of grading system (45), existence of clinical, pathological markers as well as genomic or transcriptomic signatures reported, potentially useful to guide treatment selection and predict response to systemic therapy, choosing the most optimal therapy with best treatment response at a patient-specific level is currently the biggest unmet need (46).

This is where the KATY project steps in developing a system which is interactively trained rather than being strictly programmed allowing for prediction without predefined rules and providing explainability to end users. The AI-assisted KATY Platform is built around the (i) KATY Holistic Neural Network (KATYHoNN) black-box model and the (ii) Distributed Knowledge Graph offering *post-hoc* explainability.

## Datasets and ontologies

To build the knowledge graph for the KATYHoNN model we utilize two type of datasets:

(i) data from clinical trials evaluating the efficiency of targeted therapies, immunotherapies and combination of both,
(ii) publicly-available omics datasets for ccRCC patients.

We divided these dense and multi-modal datasets into “main” and “support” datasets. The “main dataset” makes use of (i) data from clinical trials and associated ontologies to address the question on the treatment effectiveness measured for each antitumor drug and estimate response-to-treatment metrics [i.e., tumor shrinkage, overall response rate (ORR), progression-free survival (PFS), and overall survival (OS)].

The “support dataset” makes use of (ii) publicly-available omics datasets and associated ontologies to predict features which are not directly linked to response-to-therapy.

**Figure 1:**
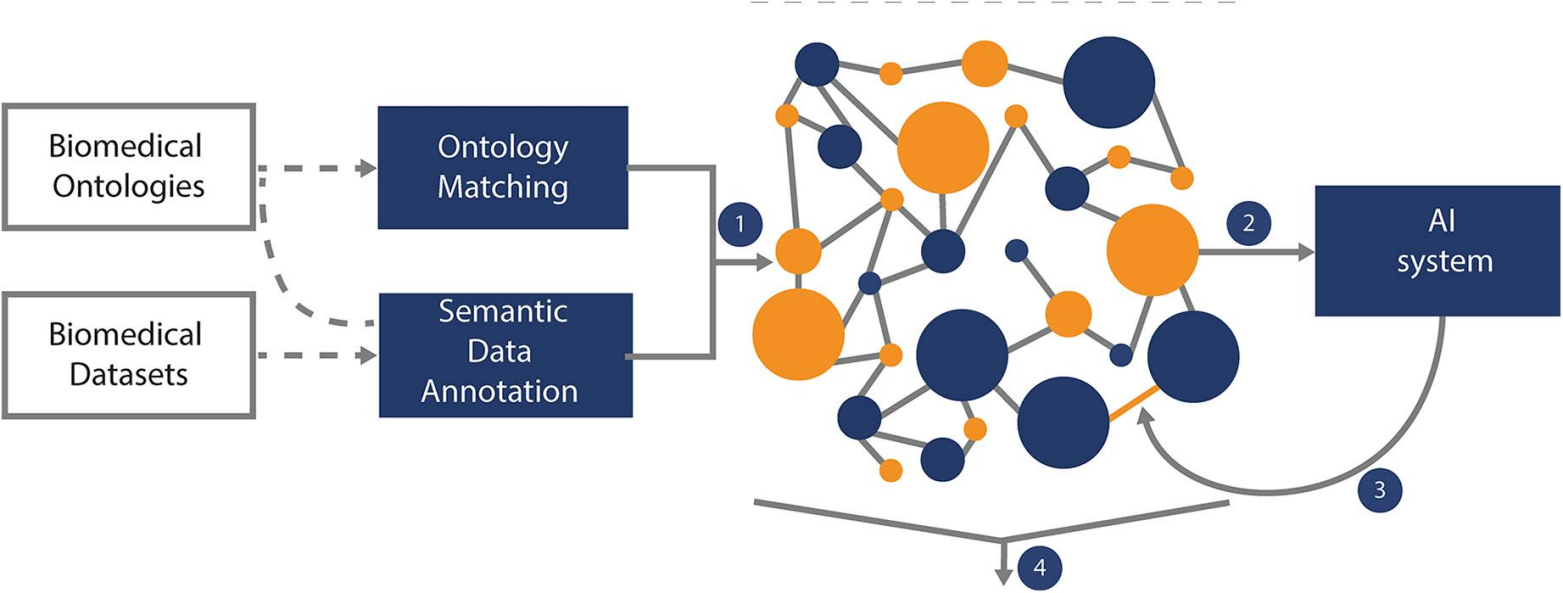
Knowledge graph construction and design. The Knowledge Graph is built using techniques of Ontology Matching and Semantic Annotation over renal cell carcinoma ontologies and KATY datasets stored in the data lake (the Knowledge Graph is stored in a GraphDB instance that supports fast loading, querying and visualization of the graph) (1). Upon development, the Knowledge Graph can be used as a source of knowledge-enriched features for the AI system (2). The outcomes of the AI-supported system can refine the Knowledge Graph (3). The data and outcomes as represented in the Knowledge Graph can be used to support explanation generation (4). Querying and visualizing the Knowledge Graph is supported by intelligent graphical user interfaces to present explanations to end-users.

Both data types are integrated into a knowledge graph through a network of ontologies, to offer comprehensive, interconnected and explainable representation of treatment effectiveness and influencing factors for a single patient (Figure 1).

### KATY model

**Figure 2:**
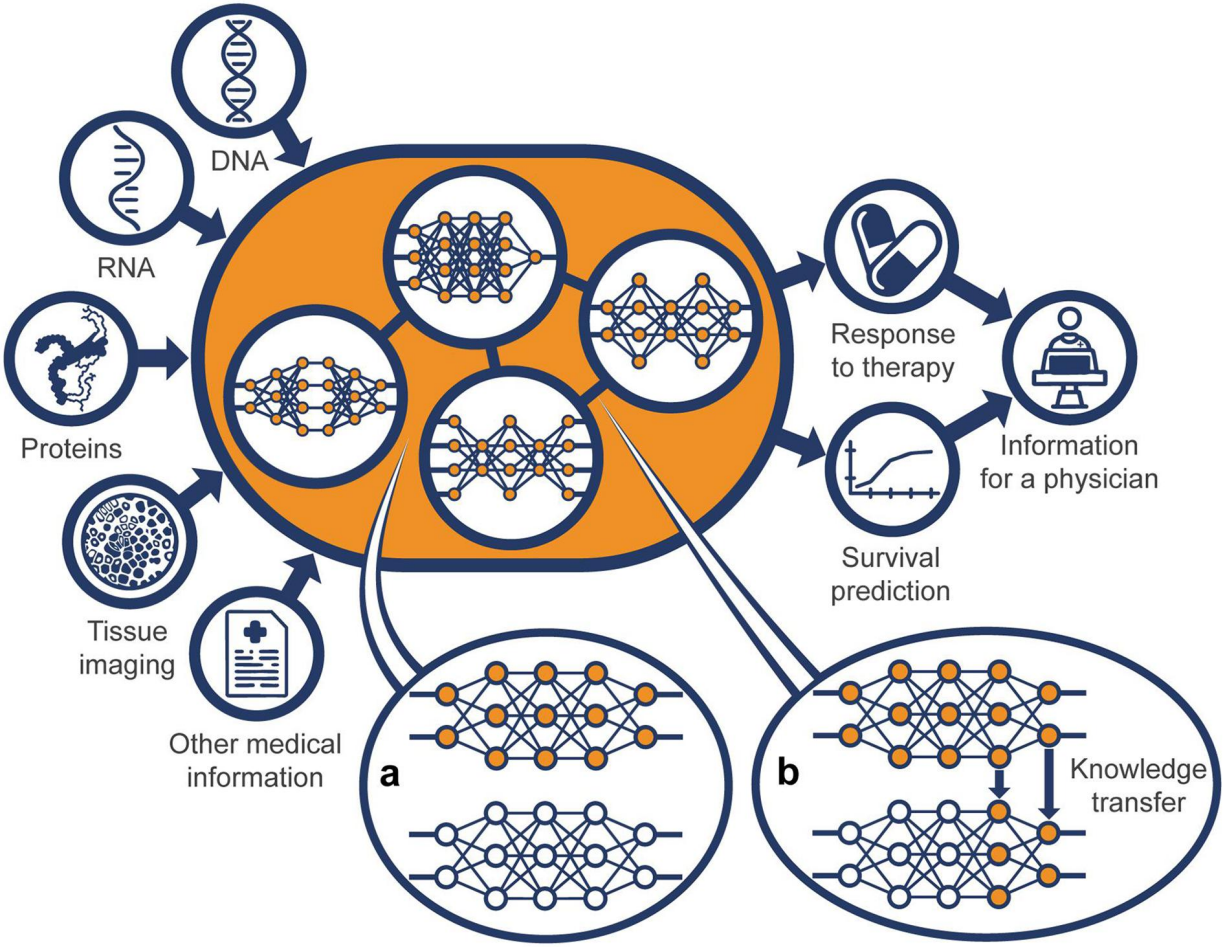
General representation of the KATY holistic neural network model (KATYHoNN). The KATYHoNN model input leverages publicly-available datasets for ccRCC patients and data from clinical trials evaluating the efficiency of therapies. The heart of the model is composed of individual sub-networks for which the input is available omics data and clinical patient data. The sub-networks can be trained either on: (i) singular specific task (e.g., either genomics or transcriptomics or proteomics or RNA-Seq data) via transfer learning (b), or (ii) all tasks together (patients data evaluating the efficiency of therapies) via multi-task learning (a) to compile the general network. The result of a multi-task learning (a) is prediction of response-to-therapy, while the results from transfer learning (b) is prediction of other features not directly related to treatment choice. The KATYHoNN model deployed on a dedicated KATY Platform will allow a physician to upload a patient's test results and gain the best treatment recommendation in parallel with KG-driven explanation based on which features such a treatment was proposed and what the survival rate for that patient is.

The KATY model consists of sub-networks that are trained using data from clinical trials and omics data for ccRCC patients and also more widely available -omics data, for example T-cell antigen landscapes published across over 2,300 published samples. Sub-division of the KATYHoNN model into sub-networks (trained either on specific omics dataset or on all omics datasets together) makes the realization of the final model easier and more precise, as each component is trained for a specific task and can be easily handled in the event of errors or changes. This training strategy mitigates the missing data problem, where there may be sufficient data for a specific task but insufficient data for all tasks combined. In case of insufficient data for model training, data from different cancer types can be used to pre-train specific sub-networks which can then be *transferred* to the ccRCC model. With this approach referred to as transfer learning, the KATYHoNN model can exploit future datasets enabling real-time training of the entire model. In this way the KATYHoNN model is easily modifiable, as at the same time, it is possible to add new components within the KATYHoNN model and modify the existing ones by deleting or linking them. The results from the KATYHoNN model deployed onto a dedicated KATY Platform and supported with the knowledge graph will allow physicians to predict survival/response to therapy when uploading patient's test results (Figure 2).

## Legal compliance

The KATY project ensures that the development and usage of the KATY Platform, as well as the use and re-use of the data generated and collected in the KATY project, is conducted in accordance with binding legal provisions. The project monitors and addresses the requirements set forth in the EU legal acts concerning (personal) data protection (especially with regard to special categories of data, i.e., data concerning health and genetic data), AI and data governance. Once the data protection and privacy principles have been identified, technical solutions such as data access management, encryption, cryptographic components, pseudonymisation and, where achievable, anonymisation of data, data minimisation, secure storage of data and secure data transfer, have been included in the design of the KATY Platform allowing for the European Union's General Data Protection Regulation (GDPR)-compliant processing of the data and usage of the platform.

Summing up, the KATY project develops the infrastructure for a ready-to-use explainable decision-support system that complies with all legal requirements (11).

## Discussion

To realize the vision of an AI-empowered Personalized Medicine in the clinical workflow, we need to tackle a challenge of developing a multi-tiered AI-supported system capable of handling complexity of multi-modal data simultaneously being adaptable to new knowledge and constantly changing treatment guidelines. To enhance maintainability and scalability of the system, it should accommodate 3 tiers: the Data Tier, which ensures secure storage and interoperability of diverse medical data; the Application Tier, which processes data and generates recommendations; and the Presentation Tier, which provides user-friendly interfaces for clinicians and patients. The separation between 3 tiers facilitates managing of the whole system, so that changes in one tier do not necessarily affect the other tiers (47–49).

Over the next several years, we anticipate the establishment of digital medicine centers that will invest in human resources, assembling well-connected medical, IT, and research teams to make these advancements a reality. Domain experts who are constantly supported by physicians will evolve to accommodate constantly evolving treatments, and build disease-oriented knowledge models in a domain-specific field.

With real-time explainable treatment recommendations, the next step would be to link post-treatment (follow-up) data of individual patients with potential side effects and drug interactions, personalized to their medical history and current medications. To fully leverage AI-supported systems linked to knowledge-based models, we must start collecting omics data for each patient within the healthcare system while simultaneously training and motivating doctors on how and why to use these systems in their daily practice. In the future, those who incorporate AI-supported tools into their routine will probably replace those who do not.

Encouraging transparent communication about the complexity and data sparsity in biomedical AI areas, like personalized cancer treatment, is essential for aligning funding institutions and stakeholders on feasible solutions. Valuable clinical trial data and -omic data is hidden by numerous legal agreements, creating a significant barrier to integrating and making this data accessible for AI systems. In these clinical trial contexts, patient health is prioritized over data volume, leading to the use of simpler, transparent models developed to address specific clinical questions. A modern approach may involve leveraging foundation models—large, pre-trained AI models for specific data types that can be fine-tuned for various tasks—and using them as black-box models with *post-hoc* explainability rooted in visual representation and feature relevance.

Despite efforts to build AI-supported decision systems, a systematic study of explanations for decisions within disease-specific domains is still missing. The explanations offered by AI decision-support systems must provide understandable and conceptually coherent knowledge. Only then can AI models provide transparent, human-interpretable explanations of AI-assisted diagnosis and treatment recommendations, facilitating their adoption in real clinical settings. Building on how the AI model explains its predictions is important for gaining trust from the medical community and establishing domain-expert interpretation and inference grounded in current medical findings. Addressing these gaps in explanation structure and knowledge leveraging is essential for advancing knowledge-based model development driving explainable AI.

We believe that continuous interaction between physicians and AI provided by the system's architecture, along with dynamically curated databases and ongoing feedback from doctors, is essential for ensuring evidence-based, data-driven and research-supported recommendations for specific disease areas. However, we also recognize that without real-world outcome data—such as predicted or observed survival curves for patients across different treatment regimens—these systems are unlikely to significantly impact clinical decision-making. This challenge is particularly important in rare cancers, where data scarcity limits the generation of robust comparative insights. In this context, recent advances in reasoning-oriented large language models, such as OpenAI's o3 and o4 series, may offer a promising direction. These models are capable of integrating structured biomedical knowledge, disclosing their internal reasoning processes, and producing auditable, evidence-supported treatment rationales—potentially bridging the gap between algorithmic outputs and clinically actionable insight.

## Data Availability

The original contributions presented in the study are included in the article/Supplementary Material, further inquiries can be directed to the corresponding author.
